# *Streptococcus didelphis* infection in free-ranging white-eared opossum (*Didelphis albiventris*) and Brazilian common opossum (*Didelphis aurita*): pathology, microbiologic, and genomic characterization

**DOI:** 10.1371/journal.pone.0348357

**Published:** 2026-04-30

**Authors:** Daniel Oliveira dos Santos, Yasmin Gonçalves de Castro, André Duarte Vieira, Bruna Hermine de Campos, Lucas dos Reis de Souza, Nayara Ferreira de Paula, Vasco Azevedo, Bertram Brenig, Caio de Castro Cunha Figueiredo, Letícia Neves Ribeiro, Janaína Ribeiro Duarte, Vinícius Henrique Barbosa Amaral, Carlyle Mendes Coelho, Herlandes Penha Tinoco, Marcelo Pires Nogueira Carvalho, Ayisa Rodrigues de Oliveira, Rodrigo Otávio Silveira Silva, Renato Lima Santos

**Affiliations:** 1 Departamento de Clínica e Cirurgia Veterinárias, Escola de Veterinária, Universidade Federal de Minas Gerais, Belo Horizonte, Minas Gerais, Brazil; 2 Departamento de Medicina Veterinária Preventiva, Escola de Veterinária, Universidade Federal de Minas Gerais, Belo Horizonte, Minas Gerais, Brazil; 3 Instituto de Ciências Biológicas, Universidade Federal de Minas Gerais, Belo Horizonte, Minas Gerais, Brazil; 4 Institute of Veterinary Medicine, University of Göttingen, Göttingen, Germany; 5 Fundação de Parques Municipais e Zoobotânica, Belo Horizonte, Minas Gerais, Brazil; Instituto Oswaldo Cruz, BRAZIL

## Abstract

Marsupials of the genus *Didelphis* are highly adapted to urban environments and are widely distributed in the Americas. *Streptococcus didelphis* is a bacterium that has been isolated from, and associated with disease, in the Virginia opossum (*Didelphis virginiana*) and the white-eared opossum (*Didelphis albiventris*). This study describes pathological changes associated with *S. didelphis* infection in white-eared opossums (*Didelphis albiventris*) and Brazilian common opossums (*Didelphis aurita*) as well as microbiological and genomic characterization of isolates. Ten opossums underwent necropsy and had ulcerative dermatitis of probable traumatic origin had *S. didelphis* was isolated from cutaneous lesions (n = 16) or systemic sites (n = 4). In contrast, 34 free-ranging opossums that were captured had negative culture results for *S. didelphis* from nasal swabs. Other lesions in opossums naturally infected with *S. didelphihs* included splenitis (7/10), myocarditis (6/10), interstitial nephritis and pyelonephritis (7/10), and myositis (4/10). Most isolates were susceptible to the antimicrobial drugs tested and none of them were able to form biofilm *in vitro*. Whole genome analysis of six isolates revealed no resistance determinants, virulence factors or plasmids, and the isolates showed high genomic similarity.

## Introduction

Opossums are marsupials with generalist habits regarding diet and habitat, which favors their movement and adaptation to diverse environments. Thus, these animals are well adapted to urban areas, where they can interact with humans and domestic animals, as well as natural environments, where they interact with other wild animals [1–3]. Therefore, understanding the factors that lead to disease in these animals is important, as they can act as reservoirs of pathogens relevant to human and animal health [3–8]. Opossums are widely distributed across the American continent and the white-eared opossum (*Didelphis albiventris*) and the Brazilian common opossum (*Didelphis aurita*) are commonly found in Brazil.

The genus *Streptococcus* includes 212 species [9], characterized by gram-positive, spherical or ovoid cells, typically arranged in chains or pairs. They are found in the environment and can be commensal in both animals and humans [10]. *S. didelphis* was first described in the Virginia opossum (*Didelphis virginiana*) associated with dermatitis and septicemia [11]. More recently, *S. didelphis* infection was reported in a Virginia opossum coinfected with macropodid alphaherpesvirus 2 [12] and in a Virginia opossum with suppurative vaginitis and sepsis [13]. In the white-eared opossum, *S. didelphis*-associated infection was recently reported in only two animals with skin necrosis and pleuritis [14]. Given the scarcity of information about *S. didelphis* infection, this study aimed to describe the pathological aspects of naturally infected free-ranging white-eared and Brazilian common opossums, as well as the microbiological and genomic features of the isolates.

## Materials and methods

### Ethics statement

All procedures were previously approved by the Ethics Committee on the Use of Animals of the Universidade Federal de Minas Gerais (CEUA/UFMG) under protocol number 79/2022, by Instituto Chico Mendes de Conservação de Biodiversidade under protocol number 81680 and by Fundação de Parques Municipais e Zoobotânica de Belo Horizonte (FPMZ-BH) under protocol number FU003/2022. The study was also registered at Sistema Nacional de Gestão do Patrimônio Genético Tradicional Associado (SisGen) under protocol numbers ABB00DB and ACDCB02.

### Necropsy and histopathology

Dead opossums were found at the *Parque Américo Renné Gianetti* (AR); *Parque Aggeo Pio Sobrinho* (AP); *Universidade Federal de Minas Gerais* campus (C); *Parque Fazenda Lagoa do Nado* (LN); *Parque das Mangabeiras* (PM); *Parque Ursulina de Andrade Mello* (U) and Belo Horizonte Zoo (Z), all located in Belo Horizonte, State of Minas Gerais, Brazil (coordinates 19° 55′ 00″ S, 43° 56′ 00″ W) (S1 Fig). Free-ranging opossums submitted to necropsy underwent gross examination, and all opossums from which *S. didelphis* was isolated were included in this study. Sex was recorded for each animal and age was estimated based on animal size and dentition development. Adults had bigger body sizes and fully developed dentitions and young had smaller body size and incomplete dentition. Samples from skin (ear and tail), superficial lymph nodes, salivary glands, brain, tongue, esophagus, stomach, small and large intestines, pancreas, trachea, lungs, heart, spleen, liver, gallbladder, kidneys, urinary bladder, testis, ovaries, uterus and bone marrow were systematically sampled and fixed in 10% buffers formalin solution for histopathology. Other samples such as bone, skeletal muscle and skin from other locations were also sampled when gross lesions were observed. Gross examination focused particularly on the presence of previous trauma, skin inflammatory infiltrate, extent of the lesions, systemic findings, and intravascular bacterial aggregates suggesting bacteremia. Formalin-fixed samples were routinely processed for paraffin embedding and 2–3 µm slides were then stained with hematoxylin and eosin or Gram stain. Based on gross examination, samples (swabs or tissue samples) from animals with suspected bacterial infection were collected for isolation.

### Field procedures

In addition to pathological and microbiological analyses of opossums that were found dead, free-ranging opossum were captured in six urban parks in the city of Belo Horizonte (*Parque das Mangabeiras* – PM, *Parque da Serra do Curral* – SC, *Parque Ursulina Andrade de Mello* – U, *Parque Aggeo Pio* – AP, *Parque Fazenda Lagoa do Nado* – LN and Belo Horizonte Zoo – Z) with Tomahawk traps baited with bananas. Captured animals were physically and chemically restrained (Ketamine 10 mg/Kg and Midazolam 1 mg/Kg) prior to clinical evaluations and sample collection. Nasal swabs were sampled from all animals, and skin swabs were also sampled from individuals with skin lesions. All swabs were submitted to bacterial isolation. All captured animals were included in the study, identified with microchips, and released after sedation recovery.

### Bacterial isolation and identification

Swabs were plated on brain heart infusion agar (BHI, Kasvi, Brazil) supplemented with 5% equine blood and MacConkey agar (BHI, Kasvi, Brazil) and incubated at 37°C for 48 h under aerobic conditions. Isolates were subjected to species identification by matrix-assisted laser desorption/ionization-time-of-flight mass spectrometry (MALDI-ToF MS; Bruker Daltonics, USA). Analyses were performed with pure colonies according to the manufacturer’s instructions, considering a confidence score ≥ 2.300 for species-level identification [15].

### Antimicrobial susceptibility tests

Antimicrobial susceptibility of all *S. didelphis* isolates was evaluated by disk diffusion method [16]. Seven antimicrobials from five classes were tested: ampicillin (AM, 10 µg), cefotaxime (CTX, 30 µg), clindamycin (CM, 2 µg), chloramphenicol (CL, 30 µg), erythromycin (ERY, 15 µg), penicillin (PEN, 10 µg), and tetracycline (TET, 30 µg). The results were interpreted according to the criteria established in the CLSI [16] for *Streptococcus* spp. of the β-hemolytic group.

### Biofilm formation test

Biofilm production capacity of the isolates was performed in microtiter plates according to Stepanović et al. [17]. Samples of *Staphylococcus epidermidis* ATCC 35984 (SE35984) and *Staphylococcus aureus* ATCC 6538 (SA6538) were used as positive control, while wells containing Tryptic Soy Broth (TSB, Oxoid, United Kingdom) were used as negative control. The reading was performed in a spectrophotometer (492 nm) (Thoth, Brazil).

### DNA extraction and genomic sequencing

Six isolates from different cases underwent whole-genome sequencing. Strains were incubated on brain-heart infusion agar (BHI, Kasvi, Brazil) supplemented with 5% equine blood at 37°C for 48 h. Genomic DNA was extracted using the Wizard Genomic DNA Purification Kit (Promega, USA). Genome sequencing was performed using the Illumina MiSeq platform with the MiSeq Reagent Kit v2, generating paired-end reads of 2 x 150 pb. The generated raw data was analyzed with FastQC (Babraham Bioinformatics, Cambridge, England), retaining only paired-end reads with a Phred score ≥ 30. Assembly was performed using the de novo method with SPAdes v3.13.0 in careful mode [18,19]. Gaps were filled with Pilon [20]. Genomic sequences were submitted to the GenBank (https://www.ncbi.nlm.nih.gov/genbank/) under BioProject ID: PRJNA1265247. Individual sample identifiers are detailed in Table 1.

**Table 1:** *Streptococcus didelphis* isolates and genome submission details.

Sample	ID	BioSample	Organism	Genome size (bp)
**SD01**	1	SAMN48590870	*S. didelphis*	1.84 Mb
**SD02**	3	SAMN48590871	*S. didelphis*	1.85 Mb
**SD03**	11	SAMN48590872	*S. didelphis*	1.84 Mb
**SD04**	4	SAMN48590873	*S. didelphis*	1.84 Mb
**SD05**	5	SAMN48590874	*S. didelphis*	1.84 Mb
**SD06**	16	SAMN48590875	*S. didelphis*	1.85 Mb

### Genomic analysis

Bacterial species prediction from genomes was performed using Kmerfinder 3.2 [21]. ResFinder 4.1 [22] was used to identify the determinants of acquired antimicrobial resistance, adopting minimum criteria of 90% identity and 60% coverage. The search for virulence factors was performed using ABRicate, using the VFDB (Virulence Factors of Pathogenic Bacteria) database [23]. Single nucleotide polymorphism (SNP) analysis was performed using CSIPhylogeny 1.4 [24] with a minimum Z-score of 1.96 and a minimum depth at the SNP position of 10x. For this analysis, the genome of *S. didelphis* DSM 15616 (Accession number: SAMN02256413) was used as a reference, and the genomes of LBVP100_21 (Accession number: SAMN30684696) and LBVP101_21 (Accession number: SAMN30684697) were included for genetic comparison purposes. The phylogenetic tree was visualized using the online software iTOL applying automatic midpoint rooting [25].

## Results

### Animals

From July 2021 to April 2025, ten free-ranging white eared opossums were submitted to necropsy and diagnosed with *S. didelphis* infection based on bacterial isolation and pathologic findings. All animals were adults, three females and seven males (Table 2).

**Table 2:** General data of necropsied and live captured free-ranging white-eared opossums (*Didelphis albiventris*) and Brazilian common opossums (*Didelphis aurita*) in which *Streptococcus didelphis* was isolated from tissues.

ID	Species	Sex	Estimated age	Origin^*^	Sampling year
**Necropsied opossums**					
**1**	*D. albiventris*	Male	Adult	AR	2021
**2**	*D. albiventris*	Male	Adult	AR	2021
**3**	*D. albiventris*	Female	Adult	AR	2021
**4**	*D. albiventris*	Male	Adult	AR	2022
**5**	*D. albiventris*	Male	Adult	LN	2022
**6**	*D. albiventris*	Male	Adult	C	2022
**7**	*D. albiventris*	Male	Adult	Z	2023
**8**	*D. albiventris*	Male	Adult	Z	2023
**9**	*D. albiventris*	Female	Adult	Z	2025
**10**	*D. albiventris*	Female	Adult	C	2025
**Captured opossums**					
**11**	*D. aurita*	Female	Young	PM	2022
**12**	*D. albiventris*	Female	Adult	U	2023
**13**	*D. albiventris*	Female	Adult	U	2023
**14**	*D. albiventris*	Female	Young	LN	2023
**15**	*D. albiventris*	Female	Adult	Z	2023
**16**	*D. albiventris*	Male	Adult	LN	2023
**17**	*D. albiventris*	Female	Adult	LN	2023
**18**	*D. albiventris*	Male	Adult	LN	2023
**19**	*D. albiventris*	Male	Adult	AP	2023
**20**	*D. albiventris*	Female	Adult	LN	2023

* AR: *Parque Américo Renné Gianetti*; AP: *Parque Aggeo Pio Sobrinho*; C: *Universidade Federal de Minas Gerais* campus; LN: *Parque Fazenda Lagoa do Nado*; PM: *Parque das Mangabeiras*; U: *Parque Ursulina de Andrade Mello*; Z: Belo Horizonte Zoo.

**Fig 1:**
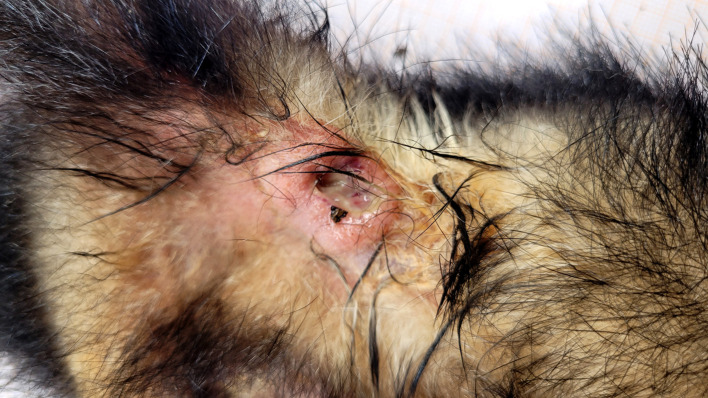
*Streptococcus didelphis*-associated ulcerative dermatitis, skin, *Didelphis aurita.* Locally extensive area of ulceration with elevated borders and central purulent exudate.

Additionally, free-ranging live opossums were captured in six urban parks to investigate *S. didelphis* as a part of nasal microbiota and possible association with skin lesions. Nasal swabs from 34 opossum (32 white-eared opossums and two Brazilian common opossums) were submitted to bacterial isolation and no samples were positive for *S. didelphis*. However, ten of the 34 captured free-ranging opossums presented skin lesions, consisting of varying degrees of ulceration and purulent exudate (Fig 1), from which *S. didelphis* was isolated. Eight of the *S. didelphis* positive opossum were adults and the other two were young. Seven of them were females and three were males. Only one of the ten positive animals was a Brazilian common opossum, the nine remaining positive animals were white-eared opossum. Table 2 describes general data (species, sex, estimated age, origin of the animal and year of sampling) from all *S. didelphis* positive opossum, both necropsied and captured.

### Pathological findings

**Fig 2:**
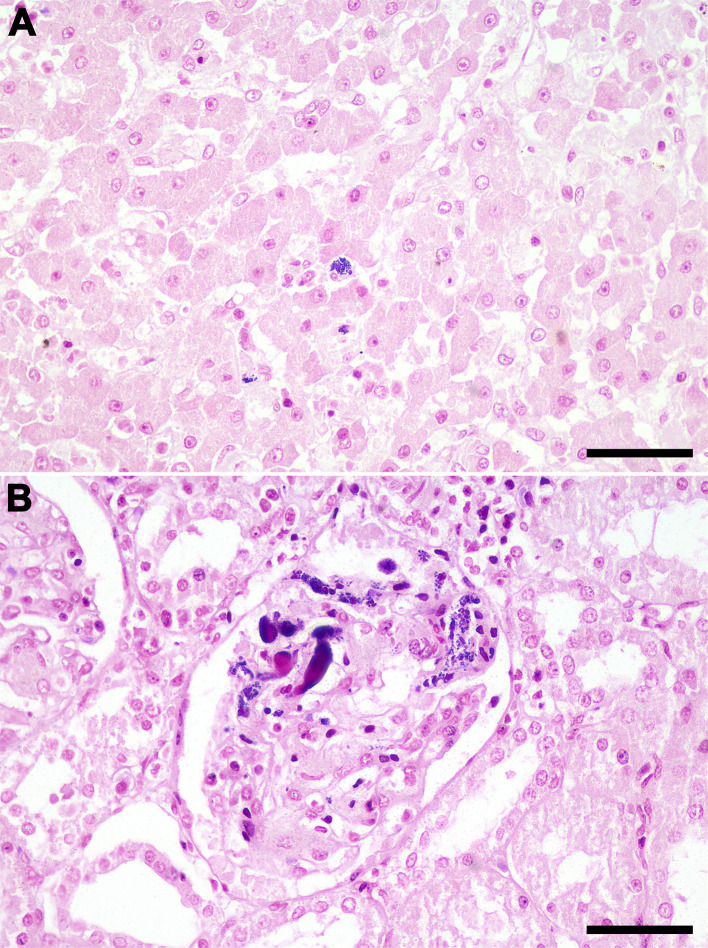
*Streptococcus didelphis* bacteremia. **(A)** Mild multifocal gram-positive cocci intravascular in sinusoids and intracytoplasmic in Kupffer cells, liver, *Didelphis albiventris*, Gram stain; bar = 50 μm. **(B)** Moderate multifocal gram-positive cocci intravascular in glomeruli capillaries, kidney, *Didelphis albiventris*, Gram stain; bar = 50 μm.

**Fig 3:**
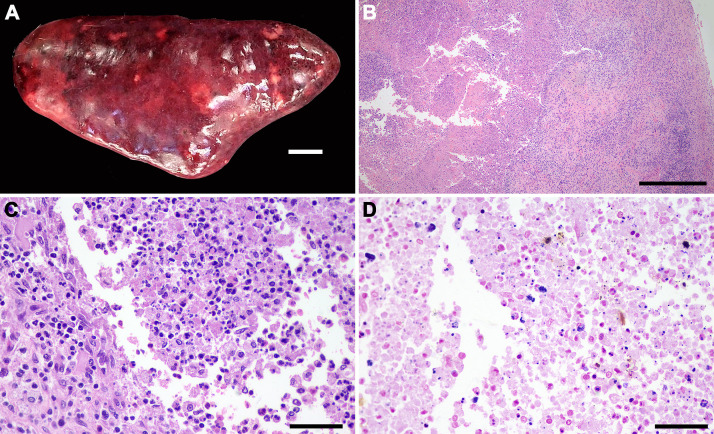
*Streptococcus didelphis*-associated splenic lesions in *Didelphis albiventris.* **(A)** Severe diffuse splenomegaly with multifocal to coalescent white areas, bar = 1 cm. **(B)** Locally extensive necrosis delimited by fibrosis, HE; bar = 500 μm. **(C)** Necrosis with neutrophilic infiltrate, HE; bar = 50 μm. **(D)** Multifocal gram-positive cocci in an area of necrosis, Gram stain; bar = 50 μm.

**Fig 4:**
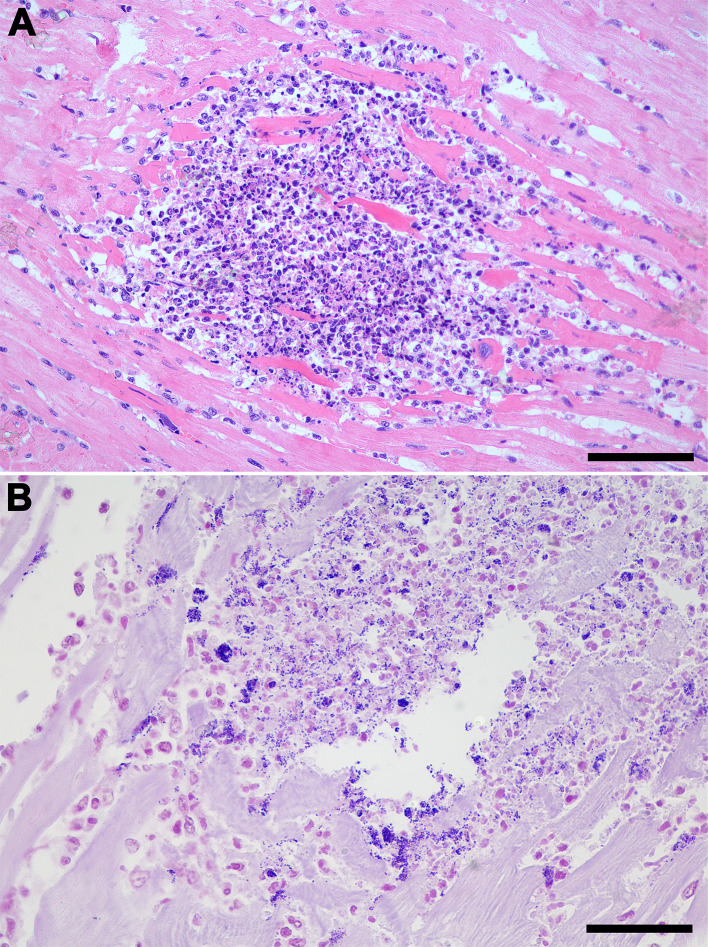
*Streptococcus didelphis*-associated cardiac lesions in *Didelphis albiventris.* **(A)** Moderate focal loss of cardiomyocytes and neutrophilic infiltrate, heart, HE; bar = 100 μm. **(B)** Gram-positive cocci associated with neutrophilic myocarditis, Gram stain; bar = 50 μm.

**Fig 5:**
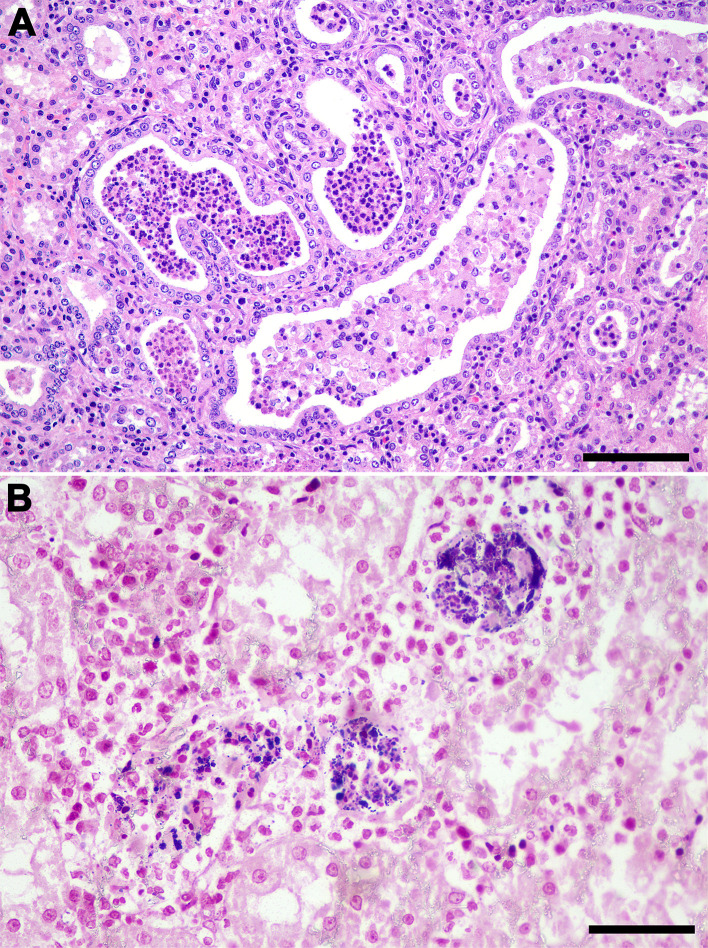
*Streptococcus didelphis*-associated renal lesions in *Didelphis albiventris.* **(A)** Multiple tubules with intraluminal neutrophilic infiltrate (pyelonephritis), HE; bar = 100 μm. **(B)** Gram-positive cocci associated with pyelonephritis, Gram stain; bar = 50 μm.

**Fig 6:**
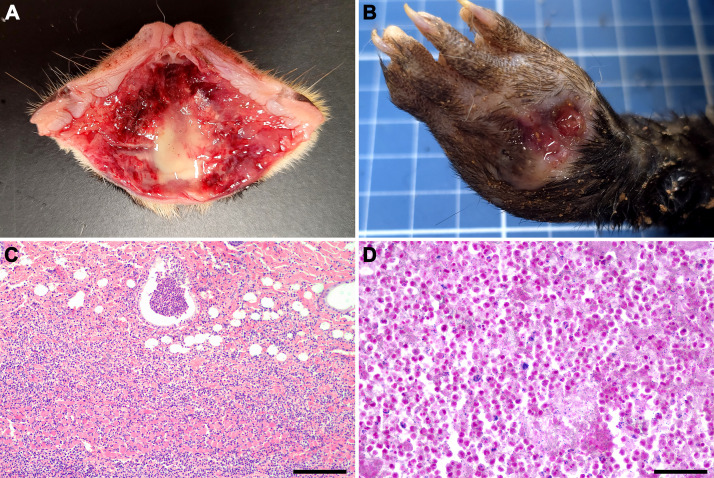
*Streptococcus didelphis*-associated cutaneous lesions in *Didelphis albiventris.* **(A)** Subcutaneous tissue of the nasal planum with purulent exudate on the cut surface. **(B)** Focal ulcer with purulent exudate in the skin of the distal thoracic limb. **(C)** Dermis and subcutaneous with diffuse neutrophilic infiltrate, HE; bar = 100 μm. **(d)** Gram-positive cocci associated with cutaneous neutrophilic infiltrate, Gram stain; bar = 50 μm.

**Fig 7:**
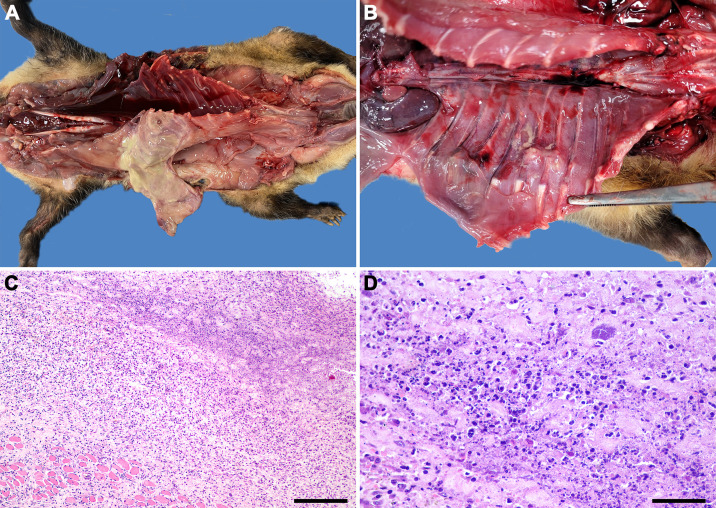
*Streptococcus didelphis*-associated muscular lesions in *Didelphis albiventris.* **(A)** Locally extensive purulent exudate between external and internal layers of skeletal muscle in the thoracic wall. **(B)** Multiple complete rib fractures with purulent exudate and hemorrhage. **(C)** Severe diffuse neutrophilic myositis, HE; bar = 200 μm. **(d)** Higher magnification of neutrophilic infiltrate in the skeletal muscle with intralesional aggregates of coccoid bacteria, HE; bar = 50 μm.

The S1 Table describes pathological findings associated with *S. didelphis* infection in the 10 necropsied opossum. Most animals (9/10) had gross lesions attributed to trauma, all of them compromised the skin integrity (lacerations or perforations). Bacteremia in these cases was characterized by the presence of bacteria intravascularly in multiple organs (Fig 2) and was observed in nine out of the 10 necropsied opossum. Most lesions were neutrophilic and necrotizing. Necrotizing and/or neutrophilic splenitis were observed in seven opossums, often associated with intralesional Gram-positive cocci (5/7) (Fig 3). Myocarditis was found in six opossums, mainly neutrophilic in most cases (5/6) it is also associated with lymphocytes and histiocytes, and in three cases with intralesional Gram-positive cocci (Fig 4). Interstitial nephritis and pyelonephritis were observed in seven animals, five of them with intralesional Gram-positive cocci (Fig 5). Cutaneous lesions were ulcerative and neutrophilic (4/10) and extended deeply to the subcutaneous tissues (Fig 6). Skeletal muscles were affected in four cases (Fig 7). In three of them large amounts of suppurative exudate were observed in the subcutaneous tissues adjacent to skin perforations (most likely due to predation). In one case (animal 2), myositis was associated with multiple rib fractures and pyothorax. All morphologic diagnosis of the necropsied opossum including lesions not associated with *S. didelphis* infection are described in the S1 Table.

### Isolation, identification, and antimicrobial susceptibility of *Streptococcus didelphis*

*Streptococcus didelphis* was isolated from many organs. Table 3 describes the isolation source and isolated bacteria from tested samples. From necropsied animals *S. didelphis* was mostly isolated from skin samples (6/10) as was for all captured animals (10/10).

**Table 3:** Source of isolates and bacterial species isolated from samples of *Streptococcus didelphis-*positive opossums.

Animal ID	Organ/tissue	Isolated bacteria	*S. didelphis* isolate ID
Necropsied opossums			
1	Liver, spleen and kidney	*Streptococcus didelphis*	Z845
2	Thoracic cavity	*Aeromonas hydrophila; Streptococcus didelphis*	Z905/21
3	Lung	*Streptococcus didelphis; Proteus mirabilis*	21073 A
4	Skin/subcutaneus (inguinal)	*Streptococcus didelphis*	B65/22
5	Skin/subcutaneous (nasal planum)	*Streptococcus didelphis*	B69/22
6	Spleen	*Streptococcus didelphis*	B125/22
7	Skin/subcutaneous (thoracic limb digit)	*Streptococcus didelphis; Mammaliicoccus sciuri; Morganella morganii*	B84/23 1
8	Skin/subcutaneous (thoracic limb), skeletal muscle (abdominal)	*Streptococcus didelphis*	B162/23
9	Skin/subcutaneous and skeletal muscle (thoracic and abdominal)	*Streptococcus didelphis*	B15/25 1M
10	Skin/subcutaneus (thoracic limb)	*Streptococcus didelphis*	B106/25
Captured opossums			
11	Skin (abdominal)	*Streptococcus didelphis*	PS30/22
12	Skin (tail)	*Streptococcus didelphis*	PS75/23
13	Skin (submandibular)	*Streptococcus didelphis*	PS76/23
14	Skin (thoracic limb)	*Streptococcus didelphis*	PS81/23
15	Skin (perinasal)	*Streptococcus didelphis*	PS106/23
16	Skin (pelvic limb)	*Streptococcus didelphis*	PS110/23
17	Skin (submandibular)	*Streptococcus didelphis*	PS113/23
18	Skin (nasal planum)	*Streptococcus didelphis*	PS114/23
19	Skin (thoracic limb)	*Streptococcus didelphis*	PS124/23
20	Skin (pelvic limb)	*Streptococcus didelphis*	G084LN

**Fig 8:**
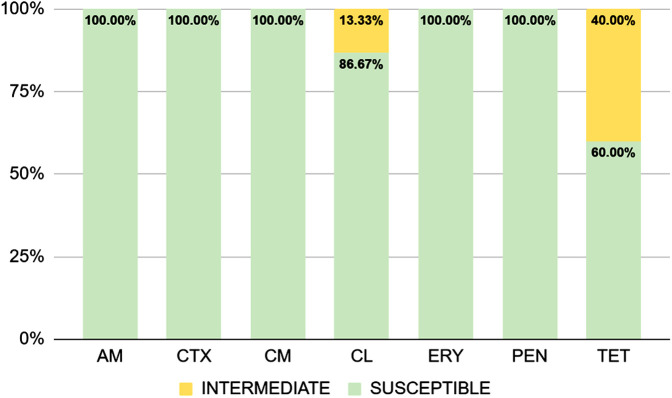
Antimicrobial susceptibility profile of *Streptococcus didelphis* isolates, obtained by disk diffusion. AM, ampicillin; CTX, cefotaxime; CM, clindamycin; CL, chloramphenicol; ERY, erythromycin; PEN, penicillin; TET, tetracycline.

**Fig 9:**
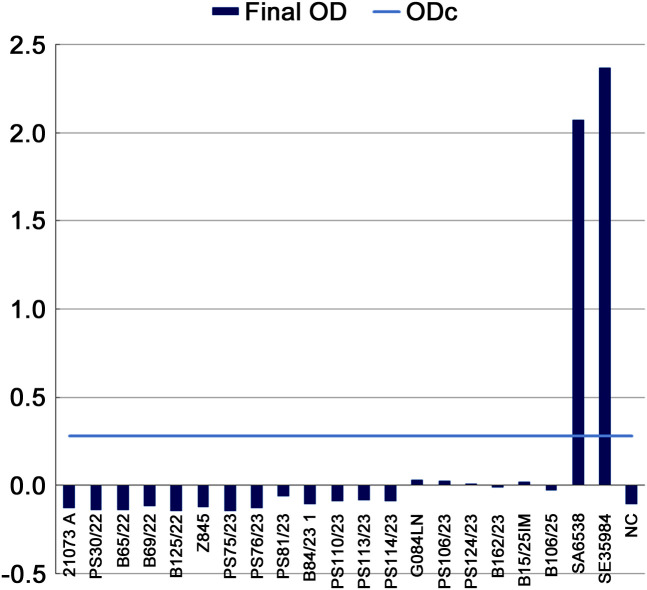
Quantification of biofilm formation by *Streptococcus didelphis* in microplates, measured by optical density (OD492 nm). ODc – optical density cut-off value for biofilm production. *Staphylococcus aureus* ATCC 6538 (SA6538) and *Staphylococcus epidermidis* ATCC 35984 (SE35984) were used as positive controls. NC: negative control.

Most of the *S. didelphis* isolates were sensitive to most of the antimicrobial drugs tested (Fig 8). None of the isolates were able to form biofilm *in vitro* (Fig 9).

### Genomic comparative analysis

**Fig 10:**
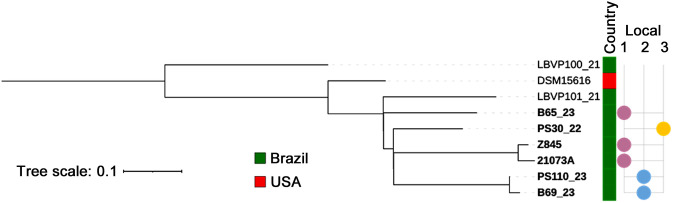
SNP-based phylogenetic tree including isolates from the present study (in bold) and reference strains for comparison.Local: 1 = *Parque Américo Renné Gianetti* (purple); 2 = *Parque Fazenda Lagoa do Nado* (blue); and *Parque das Mangabeiras* (orange). Geograpic location of these parks is described in S1 Fig.

No genetic resistance determinants, virulence factors, or plasmids were identified in the isolates from this study. SNP analysis revealed high genomic similarity among some isolates from the same location (Fig 10): Isolates from cases 1 and 3 differed by only five SNPs, while isolates 5 and 16 differed by two SNPs.

## Discussion

Here we describe several cases of *S. didelphis* infection in free-ranging opossums with pathological, microbiological and genomic characterization. Species of the genus *Streptococcus* are responsible for a wide range of diseases affecting various species, including strangles, mastitis, streptococcal meningitis, pneumonia, endocarditis, sepsis, and skin infections [26–30]. Suppurative lesions and abscess formation have frequently been observed in previous reports [31–33]. *S. didelphis* has been isolated from the Virginia opossum (*D. virginiana*) [11–13] and the white-eared opossum (*D. allbiventris*) [14]. Here, *S. didelphis* was also isolated from the Brazilian common opossum.

In this study, *S. didelphis* infection in necropsied animals affected several organs and frequently presented with neutrophilic and necrotizing lesions accompanied by bacteremia. Most necropsied opossums had traumatic lesions compromising skin integrity, which most likely represented an entry point for *S. didelphis* infection. Bacteremia was a significant feature of *S. didelphis* infection in our cases, as also described in previous studies in Virginia opossums and white-eared opossums [11,13,14]. We also observed lesions in other organs, including myocarditis and pyelonephritis, in most animals, which were associated with intralesional Gram-positive bacteria. In live-captured opossums, *S. didelphis* was isolated from 10 animals with cutaneous ulcerative and purulent lesions, supporting the hypothesis that skin lesions can serve as an entry point for infection that may progress to systemic disease.

As expected, most isolates in this study were sensitive the majority of antimicrobials tested and no resistance genes were identified in the sequenced isolates, corroborating the phenotypic results obtained in this study, and the first report of the species [11]. These findings contrast with those of a recent study, in which resistance genes to β-lactams, fluoroquinolones, lincosamides, and macrolides were detected in *S. didelphis* isolates [14]. The absence of resistance genes and the high sensitivity observed may reflect a low level of exposure of opossums to antimicrobials, especially in less urbanized areas, such as the ecological parks from which most of the samples in this study were obtained.

Although biofilm formation capability is an important virulence factor in several *Streptococcus* spp. [34], such as *S. mutans* [35], *S. pneumoniae* [36], *S. agalactiae* [37], *S. suis* [38], and *S. pyogenes* [39], its absence in *S. didelphis* does not exclude its pathogenic potential, as other mechanisms may be involved in the pathogenesis of the observed infections. The presence of the bacterium in different sites in this study reinforces this possibility and indicates the need for further research on its virulence factors. Furthermore, the absence of virulence genes in the sequenced isolates may be related to the scarcity of genomic data for the species and limited annotation in databases, which hinders the identification of genetic determinants associated with its pathogenicity.

A limitation of this study is that we were unable to define the source of infection. *Streptococcus* spp. are well-recognized commensals of mucosal surfaces, particularly the oral cavity and upper respiratory tract, where they commonly establish stable colonization [40]. Thus, we hypothesized that the likelihood of detecting *S. didelphis* in swabs from these sites would be higher. In contrast, the presence of *Streptococcus* spp. on the skin is generally considered transient and often reflects contamination from mucosal sources rather than true colonization. Moreover, the cutaneous microbiota is predominantly composed of other genera, including *Staphylococcus* and *Corynebacterium* [41], which, in the absence of selective media for *Streptococcus* spp., may further reduce the likelihood of isolating *Streptococcus* colonies, thereby decreasing detection sensitivity.

Genomic analysis of the isolates from this study has advanced our knowledge of *S. didelphis*, as available data on this species remain scarce. The low variation observed in the SNP analyses of two pairs of samples from *Parque Américo Renné Giannetti* (1 and 3) and the *Parque Fazenda Lagoa do Nado* (5 and 16) suggests the possible circulation of the same clone in these locations. In a study also conducted in Brazil, high phylogenetic similarity was observed between clinical and reference strains of *S. didelphis*, but without evidence of clonality [14], in contrast to the results of our study. These findings reinforce the importance of molecular surveillance, even in the absence of resistance or virulence genes.

This study expands knowledge of *S. didelphis* infection in free-ranging opossums, focusing on its pathological, microbiological and genomic aspects. A wide range of lesions was described in association with *S. didelphis* infection in free-ranging opossums; however, cutaneous lesions appear to be an important early feature of the disease. The high antimicrobial susceptibility, together with the absence of resistance genes, suggests a low impact from selective pressure in the environments studied. In addition, the absence of virulence factors highlights the need for studies addressing the pathogenicity mechanisms of this bacterium. Genomic analysis suggests the circulation of clones and reinforces the importance of molecular surveillance in monitoring pathogens relevant to wild animals.

## Supporting information

S1 Fig Geographic location of areas of capture or dead opossum in Belo Horizonte (Minas Gerais, Brazil). Map of the city of Belo Horizonte indicating: Belo Horizonte Zoo (Z); *Parque Fazenda Lagoa do Nado* (LN); *Parque Ursulina de Andrade Mello* (U); *Universidade Federal de Minas Gerais* campus (C); *Parque Américo Renné Gianetti* (AR); *Parque das Mangabeiras* (PM); and *Parque Aggeo Pio Sobrinho* (AP). Insets: bottom, Brazilian map indicating the State of Minas Gerais; middle, State of Minas Gerais indicating the area of Belo Horizonte; top, map of Belo Horizonte.
(TIF)

S1 Table Morphologic diagnosis of *Streptococcus didelphis*-associated lesions in naturally infected white-eared opossum (*Didelphis albiventris*). (DOCX)
