# Supplementary figures and images for: Streptococcus didelphis infection in free-ranging white-eared opossum (Didelphis albiventris) and Brazilian common opossum (Didelphis aurita): pathology, microbiologic, and genomic characterization
Source: PLoS One. 2026 Apr 30;21(4):e0348357. doi: 10.1371/journal.pone.0348357 (PMC13132194; doi:10.1371/journal.pone.0348357)

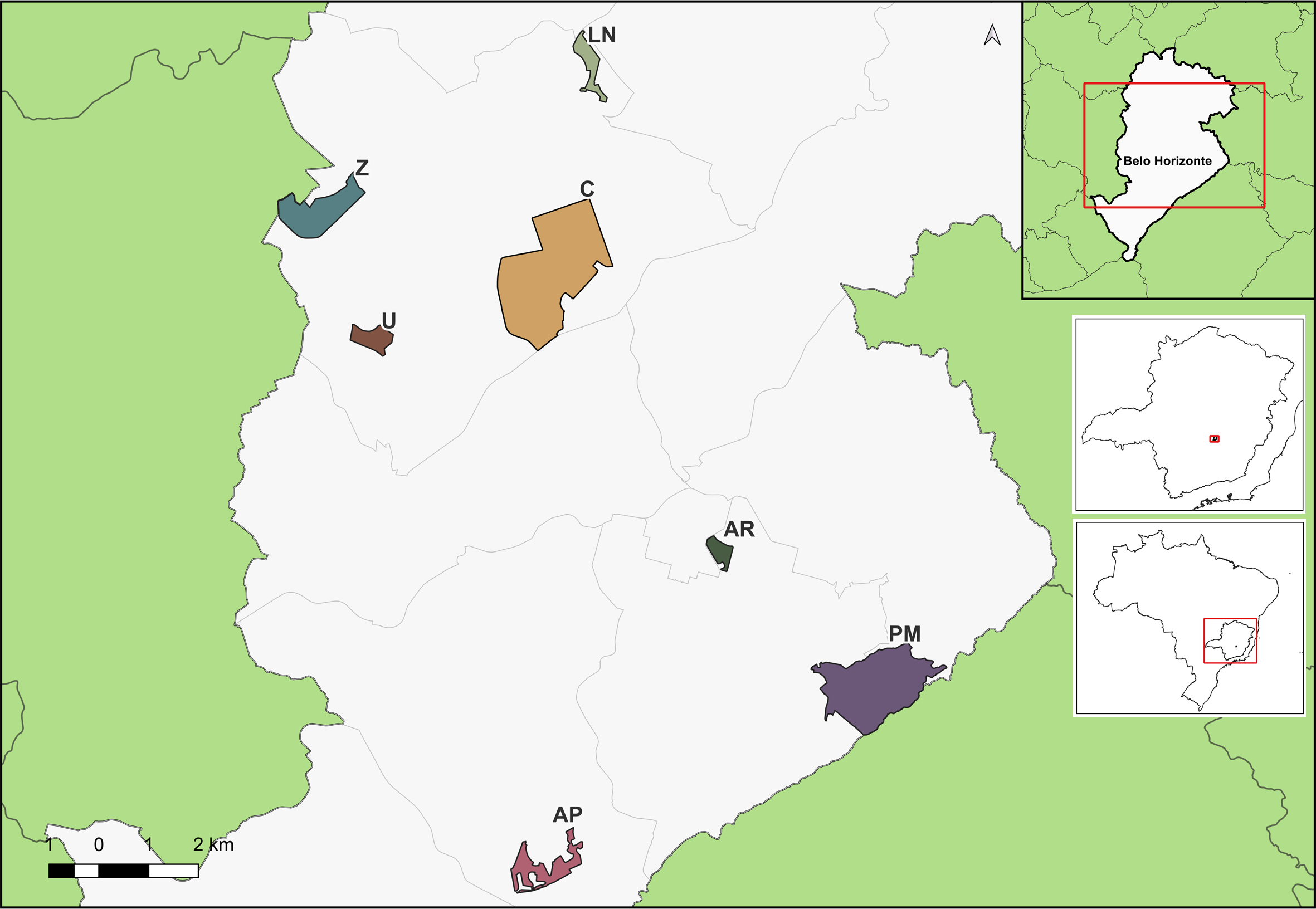

Supplement: S1 Fig — Map of the city of Belo Horizonte indicating: Belo Horizonte Zoo (Z); Parque Fazenda Lagoa do Nado (LN); Parque Ursulina de Andrade Mello (U); Universidade Federal de Minas Gerais campus (C); Parque Américo Renné Gianetti (AR); Parque das Mangabeiras (PM); and Parque Aggeo Pio Sobrinho (AP). Insets: bottom, Brazilian map indicating the State of Minas Gerais; middle, State of Minas Gerais indicating the area of Belo Horizonte; top, map of Belo Horizonte. (TIF) [file pone.0348357.s001.tif]
