# Supplementary material for: Streptococcus didelphis infection in free-ranging white-eared opossum (Didelphis albiventris) and Brazilian common opossum (Didelphis aurita): pathology, microbiologic, and genomic characterization
Source: PLoS One. 2026 Apr 30;21(4):e0348357. doi: 10.1371/journal.pone.0348357 (PMC13132194; doi:10.1371/journal.pone.0348357)
Supplement: S1 Table — (DOCX) [file pone.0348357.s002.docx]

**S1 Table.** Morphologic diagnosis of *Streptococcus didelphis*-associated lesions in naturally infected white-eared opossum (*Didelphis albiventris*).

| **ID** | **Morphologic diagnosis** | **Trauma** | **Bacteremia** |
| --- | --- | --- | --- |
| **1** | Skin (tail): moderate focal ulcer and mild focal furunculosis.  Brain: mild focal suppurative encephalitis.  Heart: moderate multifocal neutrophilic histiocytic and necrotizing myocarditis with Gram-positive cocci.  Lungs: moderate multifocal neutrophilic interstitial pneumonia with intralveolar adult nematodes (compatible with *Didelphostrongylus* sp.). Moderate multifocal mucous and neutrophilic bronchitis with nematode larvae.  Liver: moderate random necrosis with mild neutrophilic infiltrate and mild intracytoplasmic Gram-positive cocci in Kupffer cells. Mild multifocal amyloidosis.  Spleen: severe multifocal to coalescent necrotizing splenitis with Gram-positive cocci. Multifocal multinucleated giant cells.  Kidney: multifocal moderate neutrophilic pyelonephritis with Gram-positive cocci and mild intravascular Gram-positive cocci in glomerular capillaries. Mild multifocal lymphoplasmacytic interstitial nephritis.  Ureter: focal obstruction and mild multifocal lymphocytic and neutrophilic ureteritis.  Testis: mild focal neutrophilic and histiocytic epididymitis with cocci.  Tongue: mild multifocal lymphocytic glossitis.  Stomach: mild multifocal granulomatous and necrotic gastritis with intraluminal adult nematodes (compatible with *Turgida turgida*).  Small intestines: mild nematode parasitism (compatible with *Turgida turgida*).  Large intestines: moderate nematode larvae parasitism.  Pancreas: moderate locally extensive necrosis with cocci.  Adrenal: focal fibrinous thrombi with bacteria and perivascular mild hemorrhage.  Lymph nodes: moderate multifocal neutrophilic lymphadenitis.  Thyroid, bone marrow, salivary gland, urethra, prostate and urinary bladder: no significant lesions. | Yes (skin ulceration with tail bone fracture and exposure) | Yes |
| **2** | Heart: moderate multifocal necrotizing lymphocytic histiocytic and neutrophilic myocarditis with cocci. Moderate multifocal fibrosis.  Thoracic cavity: moderate diffuse pyothorax.  Lungs: severe histiocytic and lymphocytic interstitial pneumonia with intraluminal adult nematodes (compatible with *Didelphostrongyluss* sp.).  Liver: mild multifocal intracytoplasmic Gram-positive cocci in Kupffer cells. Moderate lymphocytic and histiocytic random hepatitis.  Spleen: severe multifocal to coalescent necrotizing and neutrophilic splenitis with Gram-positive cocci. Severe diffuse amyloidosis.  Kidney: severe multifocal to coalescent neutrophilic lymphocytic histiocytic and necrotizing interstitial nephritis. Severe multifocal to coalescent neutrophilic pyelonephritis with Gram-positive cocci. Mild multifocal intraluminal Gram-positive cocci in glomerular capillaries. Severe diffuse membranoproliferative glomerulonephritis.  Testis: mild testicular degeneration.  Tongue: moderate multifocal necrotic, lymphocytic, histiocytic and neutrophilic glossitis.  Stomach: multifocal mild lymphocytic and histiocytic gastritis. Rare intravascular cocci.  Adrenal: mild multifocal intravascular cocci.  Thyroid: mild focal lymphocytic, histiocytic and plasmocytic thyroiditis.  Lymph nodes: severe diffuse necrotizing and neutrophilic lymphadenitis with cocci.  Rib and skeletal muscle: severe locally extensive necrotizing neutrophilic and histiocytic myositis with Gram-positive cocci and moderate multifocal fibrosis. Moderate multifocal histiocytic and neutrophilic osteomyelitis with Gram-positive cocci.  Urinary bladder and skin (ear and tail): no significant lesions.  Brain: not sampled. | Yes (rib fracture) | Yes |
| **3** | Brain: mild multifocal suppurative encephalitis with Gram-positive cocci and moderate multifocal intravascular Gram-positive cocci.  Heart: moderate multifocal neutrophilic and histiocytic myocarditis with cocci.  Trachea: severe diffuse neutrophilic histiocytic fibrinous tracheitis with Gram-positive cocci.  Lungs: severe multifocal to coalescent necrotizing neutrophilic histiocytic and fibrinous broncho interstitial pneumonia with intravascular Gram-positive cocci. Moderate multifocal granulomatous pneumonia with intralveolar adult nematodes (compatible with *Didelphostrongylus* sp.) and larvae.  Liver: mild multifocal intracytoplasmic Gram-positive cocci in Kupffer cells. Moderate diffuse amyloidosis.  Spleen: moderate multifocal necrosis with Gram-positive cocci. Mild multifocal multinucleated giant cells.  Kidney: moderate multifocal neutrophilic pyelonephritis, moderate locally extensive neutrophilic interstitial nephritis with cocci. Mild multifocal intravascular cocci.  Uterus: mild multifocal amyloidosis.  Large intestines: moderate nematode larvae parasitism.  Adrenal: mild focal lymphocytic adrenalitis.  Lymph node: severe diffuse necrosis with Gram-positive cocci. Mild multifocal neutrophilic lymphadenitis.  Humeral radioulnar joint: bilateral moderate purulent arthritis.  Stomach and small intestines: autolysis.  Bone marrow, salivary gland, pancreas, urinary bladder, thyroid, ovaries, mammary gland and tongue: no significant lesions | Yes (tail amputation) | Yes |
| **4** | Skin and subcutaneous (inguinal and perineal): severe multifocal necrotizing and neutrophilic dermatitis, myositis, and panniculitis with Gram-positive cocci.  Brain: mild multifocal intravascular Gram-positive cocci.  Heart: mild focal lymphocytic plasmocytic and neutrophilic myocarditis.  Lungs: moderate diffuse lymphocytic, plasmocytic and neutrophilic interstitial pneumonia with intralveolar adult nematodes (compatible with *Didephostrongylus* sp.). Moderate multifocal granulomatous pneumonia with nematode larvae and eggs.  Liver: moderate diffuse amyloidosis.  Spleen: moderate multifocal to coalescent necrosis.  Kidney: severe diffuse neutrophilic pyelonephritis. Moderate multifocal interstitial fibrosis.  Stomach: moderate multifocal to coalescent eosinophilic gastritis with intraluminal adult nematodes (compatible with *Turgida turgida*).  Lymph node: moderate multifocal to coalescent histiocytic and neutrophilic lymphadenitis.  Small and large intestines, pancreas and thyroid: no significant lesions. | Yes (skin perforation) | Yes |
| **5** | Skin (face): severe locally extensive neutrophilic and ulcerative dermatitis with Gram-positive cocci.  Brain: moderate diffuse non-suppurative meningitis. Mild focal non-suppurative encephalitis.  Lungs: severe multifocal to coalescent lymphocytic, histiocytic and neutrophilic interstitial pneumonia with intralveolar adult nematodes (compatible with *Didelphostrongylus* sp.). Moderate diffuse eosinophilic bronchiolitis with intraluminal nematode larvae. Severe multifocal alveolar hemorrhage.  Liver: moderate multifocal hemorrhage. Mild random lymphocytic, histiocytic and neutrophilic hepatitis. Mild diffuse lipidosis.  Spleen: mild multifocal neutrophilic splenitis.  Kidney: mild multifocal lymphocytic interstitial nephritis.  Stomach: mild multifocal granulomatous and necrotic gastritis, moderate diffuse eosinophilic gastritis and intraluminal adult nematodes (compatible with *Turgida turgida*).  Small intestines: mild multifocal eosinophilic enteritis.  Large intestines: moderate diffuse eosinophilic enteritis with intraluminal nematode.  Adrenal: mild multifocal hemorrhage.  Thyroid: mild multifocal hemorrhage.  Lymph node (submandibular): mild multifocal neutrophilic lymphadenitis.  Bone marrow, heart, pancreas, prostate, tongue, urethra and urinary bladder: no significant lesion. | Yes (skin and skeletal muscle perforations and laceration) | No |
| **6** | Brain: mild multifocal non-suppurative meningitis. Mild multifocal gliosis. Moderate multifocal intravascular cocci.  Heart: moderate multifocal intravascular cocci.  Lungs: severe diffuse lymphocytic, histiocytic, plasmocytic and neutrophilic interstitial pneumonia with severe diffuse edema.  Liver: moderate portal lymphocytic, histiocytic and plasmocytic hepatitis. Mild periportal lipidosis. moderate multifocal intravascular cocci.  Spleen: severe locally extensive necrotizing and neutrophilic chronic splenitis with Gram-positive cocci. Severe multifocal extramedullary hematopoiesis.  Kidneys: moderate multifocal intravascular cocci.  Esophagus: moderate multifocal intravascular cocci.  Stomach: mild multifocal neutrophilic gastritis. Moderate multifocal intravascular cocci.  Small intestines: moderate multifocal intravascular cocci.  Large intestines: moderate multifocal intravascular cocci.  Pancreas: moderate multifocal intravascular cocci.  Adrenal: moderate multifocal lymphocytic and plasmocytic adrenalitis. Severe multifocal hemorrhage. Moderate multifocal intravascular cocci.  Lymph nodes: moderate multifocal histiocytic and neutrophilic lymphadenitis.  Aorta, bone marrow, skin (ear and tail), testis, tongue, trachea and urinary bladder: no significant lesions. | No | Yes |
| **7** | Skin: severe multifocal ulcerative necrotizing and neutrophilic dermatitis with cocci.  Hand: severe multifocal to coalescent necrotizing and neutrophilic dermatitis, periostitis and osteomyelitis with cocci. Severe multifocal fibrinous and necrotizing arthritis.  Brain: mild multifocal intravascular Gram-positive cocci. Moderate diffuse non-suppurative meningitis.  Heart: severe multifocal lymphocytic, histiocytic and plasmocytic myocarditis.  Lungs: severe multifocal histiocytic, neutrophilic and eosinophilic interstitial pneumonia with moderate multifocal alveolar edema, intrabronchial nematode larvae, moderate focal lipid pneumonia and focal intravascular cocci.  Liver: mild multifocal intracytoplasmic Gram-positive cocci in Kupffer cells. Severe diffuse amyloidosis. Severe multifocal extramedullary hematopoiesis.  Spleen: moderate multifocal extramedullary hematopoiesis.  Kidney: severe multifocal neutrophilic pyelonephritis with Gram-positive cocci. Severe multifocal to coalescent lymphocytic interstitial nephritis.  Urinary bladder: mild multifocal histiocytic and plasmocytic cystitis.  Testis: moderate testicular degeneration.  Tongue: moderate multifocal lymphocytic, histiocytic and plasmocytic glossitis and focal foreign body granuloma.  Esophagus: multifocal intraepithelial adult nematodes (compatible with *Gongylonema* sp.).  Stomach: severe multifocal to coalescent necrotic, eosinophilic and neutrophilic gastritis with intraluminal adult nematodes (compatible with *Turgida turgida*).  Small intestine: severe multifocal necrotizing and neutrophilic enteritis with cocci and intraluminal adult nematodes, adult cestodes and nematode larvae.  Large intestine: moderate multifocal to coalescent neutrophilic enteritis with intraluminal adult nematodes.  Mesenteric lymph node: moderate multifocal eosinophilic, neutrophilic and eosinophilic lymphadenitis. Severe diffuse hemorrhage.  Adrenal: mild multifocal neutrophilic adrenalitis and moderate multifocal extramedullary hematopoiesis.  Lymph nodes (superficial): severe multifocal to coalescent granulomatous and neutrophilic lymphadenitis with cocci.  Pancreas, thyroid and trachea: no significant lesions. | Yes (skin perforation and ulceration) | Yes |
| **8** | Brain: mild multifocal intravascular Gram-positive cocci. Mild multifocal hemorrhage.  Heart: mild multifocal intravascular bacteria.  Lungs: moderate multifocal to coalescent histiocytic and neutrophilic interstitial pneumonia with intralveolar adult nematodes (compatible with *Didelphostrongylus* sp.). Mild multifocal intravascular bacteria.  Liver: severe diffuse amyloidosis. Mild multifocal intravascular bacteria.  Spleen: severe diffuse amyloidosis. Mild multifocal intravascular bacteria.  Kidney: severe locally extensive neutrophilic interstitial nephritis with bacteria. Moderate multifocal to coalescent neutrophilic pyelonephritis. Mild multifocal intravascular bacteria. Mild multifocal glomerular amyloidosis.  Stomach: mild multifocal intravascular bacteria.  Small intestine: mild multifocal eosinophilic enteritis.  Large intestine: moderate diffuse eosinophilic enteritis with nematode larvae.  Lymph nodes: moderate multifocal neutrophilic lymphadenitis with multifocal bacteria. Severe locally extensive hemorrhage with hemosiderosis.  Skeletal muscle: severe multifocal to coalescent neutrophilic and necrotizing myositis with bacteria.  Salivary gland: mild multifocal intravascular bacteria.  Esophagus, scrotum, testis, tongue and trachea: no significant lesions. | Yes (skin perforation) | Yes |
| **9** | Abdominal cavity: moderate diffuse neutrophilic and histiocytic peritonitis with cocci.  Heart: moderate multifocal to coalescent lymphocytic, histiocytic and neutrophilic myocarditis. Moderate multifocal subendocardic fibrosis.  Lungs: moderate multifocal to coalescent histiocytic and neutrophilic interstitial pneumonia with intralveolar adult nematodes (compatible with *Didelphostrongylus* sp.). Multifocal moderate neutrophilic bronquitis with intraluminal nematode larvae. Severe multifocal alveolar hemorrhage.  Liver: moderate multifocal intravascular Gram-positive cocci. Mild lymphocytic, histiocytic and plasmocytic portal hepatitis. Mild multifocal extramedullary hematopoiesis.  Spleen: moderate multifocal to coalescent extramedullary hematopoiesis.  Kidney: moderate multifocal intravascular Gram-positive cocci. Moderate multifocal neutrophilic pyelonephritis. Moderate multifocal lymphocytic, histiocytic, plasmocytic and neutrophilic interstitial nephritis. Mild focal tubular necrosis. Mild multifocal tubular mineralization.  Urinary bladder: mild multifocal intravascular bacteria. Mild multifocal lymphocytic, histiocytic, plasmocytic and neutrophilic cystitis.  Tongue: moderate multifocal lymphocytic, histiocytic and plasmocytic glossitis.  Esophagus: mild focal neutrophilic esophagitis.  Stomach: moderate multifocal necrotic and granulomatous gastritis, moderate diffuse eosinophilic gastritis with intraluminal adult nematodes (compatible with *Turgida turgida*).  Lymph nodes: moderate multifocal hemorrhage.  Salivary gland: moderate multifocal lymphocytic adenitis.  Skeletal muscle: severe multifocal to coalescent lymphohistiocytic and neutrophilic myositis with Gram-positive cocci.  Brain, ovary and trachea: no significant lesions. | Yes  (skin and skeletal muscle perforation) | Yes |
| **10** | Skin (thoracic limb): severe locally extensive ulcerative and neutrophilic dermatitis with Gram-positive cocci.  Brain: mild multifocal non-suppurative meningoencephalitis.  Heart: mild multifocal neutrophilic myocarditis.  Lungs: moderate multifocal to coalescent histiocytic and neutrophilic interstitial pneumonia. Moderate diffuse neutrophilic and mucous bronchitis. Focal moderate neutrophilic bronchopneumonia. Multifocal moderate alveolar hemorrhage and mild multifocal alveolar edema.  Liver: mild lymphocytic and plasmocytic portal hepatitis. Mild random glycogenosis.  Spleen: mild multifocal neutrophilic splenitis with moderate multifocal to coalescent hemorrhage and mild multifocal bacteria. Moderate multifocal amyloidosis.  Kidney: mild multifocal lymphocytic and plasmocytic interstitial nephritis. Mild multifocal membranous glomerulopathy.  Tongue: mild multifocal lymphocytic glossitis.  Stomach: moderate diffuse eosinophilic gastritis with multifocal necrosis and granulomatous gastritis and intraluminal adult nematodes (compatible with *Turgida turgida*).  Small intestine: mild diffuse eosinophilic enteritis.  Large intestine: moderate diffuse eosinophilic enteritis.  Adrenal: moderate multifocal to coalescent extramedullary hematopoiesis.  Lymph nodes (superficial): severe diffuse lymphoid hyperplasia and moderate multifocal extramedullary hematopoiesis.  Bone marrow, esophagus, ovary, pancreas, pituitary gland, salivary gland, thymus, thyroid, urinary bladder and uterus: no significant lesions. | Yes  (evisceration) | No |
